# Clinical validity of the Me and My School questionnaire: a self-report mental health measure for children and adolescents

**DOI:** 10.1186/1753-2000-8-17

**Published:** 2014-06-11

**Authors:** Praveetha Patalay, Jessica Deighton, Peter Fonagy, Panos Vostanis, Miranda Wolpert

**Affiliations:** 1 Department of Clinical, Educational and Health Psychology, University College London, Gower Street, London WC1E 6BT, UK; 2 Evidence Based Practice Unit (EBPU), University College London and the Anna Freud Centre, 21 Maresfield Gardens, London NW3 5SU, UK; 3 The Greenwood Institute of Child Health, Leicester University, Westcotes House, Westcotes Drive, Leicester LE3 0QU, UK

**Keywords:** Mental health, Children, Self-report, Validity, Me and My School, Screening

## Abstract

**Background:**

The Me and My School Questionnaire (M&MS) is a self-report measure for children aged eight years and above that measures *emotional difficulties* and *behavioural difficulties,* and has been previously validated in a community sample. The present study aimed to assess its clinical sensitivity to justify its utility as a screening tool in schools.

**Methods:**

Data were collected from service-users (n = 91, 8–15 years) and accompanying parent/carer in outpatient mental health services in England. A matched community sample (N = 91) were used to assess the measure’s ability to discriminate between low- and high-risk samples.

**Results:**

Receiver operating curves (area under the curve, *emotional difficulties* = .79; *behavioural difficulties* = .78), mean comparisons (effect size, *emotional difficulties* d = 1.17, *behavioural difficulties* = 1.12) and proportions above clinical thresholds indicate that the measure satisfactorily discriminates between the samples. The scales have good internal reliability (*emotional difficulties* α = .84; *behavioural difficulties* α = .82) and cross-informant agreement with parent-reported symptoms is comparable to existing measures (r = .30).

**Conclusion:**

The findings of this study indicate that the M&MS sufficiently discriminates between high-risk (clinic) and low-risk (community) samples, has good internal reliability, compares favourably with existing self-report measures of mental health and has comparable levels of agreement between parent-report and self-report to other measures. Alongside existing validation of the M&MS, these findings justify the measures use as a self-report screening tool for mental health problems in community settings for children aged as young as 8 years.

## Background

There is increasing interest in how best to get children and young peoples’ own views on their psychological state and sense of wellbeing. Whilst there are increasing numbers of child report measures for a range of psychological problems [1,2] most of these are for one particular type of problem, do not go below the age of 11 and charge to use (ibid). Measurement of mental health in children to date has typically been achieved by measures completed by other reporters. With the increasing focus on children’s perspective being important and necessary [3,4] which is reflected in policy focus on shared decision making in health services and the concept of self-defined recovery [5] there is a real need for measures that are valid and reliable for younger children. Moreover, research indicates that children as young as 7–8 years old are able reporters of their own mental health [6,7]. In community settings, particularly schools, self-report measurement supports screening for problems and early intervention [8]. A recent review of self-report general mental health measures [1] highlights the lack of self-report measures of general mental health for young people aged less than 11 years old. Additionally existing measures developed for wide-spread use in both community and clinic settings cost to use and can be impractical for large scale population based studies and routine outcome monitoring due to the financial burden associated with large-scale and/or long-term use. Hence, the development of the Me and My School questionnaire M&MS; [9] filled a necessary gap for a free-to-use, short, self-report screening measure of child mental health that was suitable to use with a wider age range of young people and covers both emotional and behavioural difficulties.

The M&MS questionnaire has been validated with children as young as eight years old, which makes it (as far as the authors are aware), the only free to use, validated, self-report screening measure of general mental health for children of that age. The measure was developed with short items and simple language to especially facilitate use with younger children [9]. The measure also translates to clinical settings as clinical thresholds of risk have been established to aid school based staff and practitioners in identifying high-risk children. Initial validation and analysis of psychometric properties revealed it to be a measure with good content validity, internal reliability, construct validity and minimal item-bias [9]. As per criteria outlined to validate questionnaires [10-12] these analyses indicated that the measure had good psychometric properties for the criteria that have been looked at. However, Deighton et al. [9] also recognised that assessing the properties of the measure in a clinical population would be essential towards establishing the screening capacity and clinical usefulness of the measure. Particularly, assessing the ability of the test to discriminate between community and clinic populations and the utility of the established cut-off scores are necessary steps in determining its utility as a screening tool [12].

The present study aims to test the ability of the measure to discriminate between a clinic and community sample (discriminant validity), assess the internal consistency of the scales in a clinic sample (internal reliability), compare it to another self-report measure (construct validity), examine cross-informant agreement with parent completed questionnaires (inter-rater reliability) and explore the correspondence between scale scores and clinical assessment.

## Methods

The current study uses a one-one matched two group design with a clinical sample and a community based sample to examine the discriminant validity of the M&MS questionnaire. Additionally, further analyses are carried out in the clinical sample to establish the internal and inter-rater reliability and construct validity of the measure in a clinic setting.

### Sample

#### *Clinic sample*

Data were collected from n = 91 (46.2% female, N = 42) children and adolescents (mean age = 12.34 years, SD = 2.03) attending two community out-patient teams from child and adolescent mental health services in an urban location in England (67% from one team and 33% from the other team). In order to allow for comparisons with existing data from a community sample service-users were excluded if they were younger than 8 years, older than 15 years or in circumstances where cases were deemed to be highly sensitive. A large proportion of the sample belonged to the White ethnic group (69.2%, N = 63) and the remaining participants were Asian (N = 8), mixed race (N = 6) or did not have a recorded ethnicity on file (N = 14).

Participants completed the questionnaire either before or after their session with the clinician in the mental health service. Parents and young people were given information about the study and asked for their consent. Participants were informed of the confidentiality of their responses and their right to decline to participate. Ethical approval for collecting these data was received from the National Health Services Research Ethics Committee in England.

#### *Community sample*

To allow for comparative analysis with a community sample, matched controls were selected from a sample of young people who had completed the questionnaire in the same year as part of a school based study (N = 863, aged 8–15 years, mean age = 11.97, SD = 1.65; female 48.9%; ethnicity 63.6% White) from 7 schools (4 primary and 3 secondary schools) in urban locations. The community sample was matched to the clinic sample to control for demographic differences between samples biasing the results. This was done because risk of mental health problems has shown to be varied based on gender, ethnicity and age [13]. A one-one matched community sample was created using propensity score matching psmatch2; [14], which allows finding exact or closely matched individuals based on selected criteria. Matching was done based on gender, ethnicity and age and resulted in a matched community sample of 91 participants (49.5% female, 68.6% White, mean age = 12.29, SD = 1.87).

Questionnaires were completed in classroom-based sessions facilitated by researchers. Consent was sought from parents via mail beforehand. All individuals received information about the study, including explanation of the confidentiality of their responses and their right to decline to participate and drop out at any time. Ethical approval for collecting these data was given by the university ethics board at University College London.

### Measures

#### *Me and My School (M&MS)*

The M&MS questionnaire [9] is a 16-item measure comprising of a 10-item *emotional difficulties* scale and a 6-item *behavioural difficulties* scale. Items in the emotional difficulties scale include ‘I feel lonely’ and ‘I worry a lot’; items in the behavioural difficulties scale include ‘I lose my temper’ and ‘I break things on purpose’. Participants respond to each item by selecting one of three options: Never, Sometimes, Always. Total scale scores are created by summing the item scores which results in a possible range of scores of 0–20 for the emotional and 0–12 for the behavioural difficulties scales, a higher score indicating more problems. In case of missing items person-mean (prorated) imputation was conducted for up to a third of items in the scales. During the validation of the measure cut-off scores with clinical significance were established resulting in a score of 10 and above indicating problems on the emotional difficulties scale (10–11 borderline, 12 + clinical) and 6 and above indicating behavioural problems on the behavioural difficulties scale (6 borderline, 7+ clinical). The original measure was developed as an online questionnaire but a paper-based version has since been developed and validated [15] which was used in the present study.

#### *Strengths and Difficulties Questionnaire (SDQ) self-report*

The SDQ self-report [16] is a self-report measure of mental health suitable for children older than 11 years. The measure consists of five five-item scales: emotional symptoms, conduct problems, hyperactivity, peer problems and prosocial. The first four scales also sum to give a total difficulties score. Items in this measure are generally longer and more complex than the items in the M&MS (e.gs I am nervous in new situations. I easily lose confidence [or] I fight a lot. I can make other people do what I want) to which participants respond on a 3-point scale (not true, somewhat true, certainly true). This questionnaire was completed by the 56 participants (57% female) in the clinic sample who were old enough (N = 56, 11+ years; mean age = 13.46, SD = 1.29).

#### *Parent SDQ*

Accompanying parents or carers were also asked to complete the parent version of the SDQ which like the self-report version is a 25 item measure with five scales [17]. The items in the parent version correspond closely to the items in the self-report version except being in third person form (e.g. Has at least one good friend). 92% (N = 84) of accompanying parents/carers completed the questionnaire (58.3% mothers, 10.7% fathers, 3.6% other and 28% not known).

#### *Clinical assessment*

Clinical assessments were made according to ICD-10 diagnosis or ICD-10 Z-code which represent factors influencing health status and service use (e.g. removal from home, emotional neglect, disability). For individuals with no diagnoses, under assessment or a Z-code, presenting problems were recorded. 54% (N = 49) had a clinical diagnosis, 35% (N = 32) had presenting problems, 33% (N = 30) had a Z-code and 7.7% (N = 7) had no recorded diagnosis, z-code or presenting problems.

Two child clinical psychologists then independently classified the diagnoses and presenting problems into groupings based on their clinical expertise and experience. The groupings used were *emotional, behavioural, emotional and behavioural* and *other*. This was then collated which resulted in a complete agreement in coding for 82% of the items and any disagreements between the two coding clinicians were resolved in a discussion to ensure there was a clear classifying system. Based on this classifying system, for example, depression and anxiety were classified as *emotional* and learning disorders, hyperactivity, autism, and tourette’s were in the *Other* category. These groupings were then applied to assign participants’ diagnoses (and in the absence of a diagnosis, their presenting problems) to these groups. This resulted in 34 individuals with *emotional*, 7 individuals with *behavioural,* 13 individuals with co-morbid *emotional and behavioural* and 25 individuals in the *other* clinical assessment grouping.

### Analysis

Analyses were carried out in four stages to specifically look at different psychometric properties of this measure. In the first stage, internal consistencies were computed to assess reliability of the scale in the clinic setting. In stage two the ability of the M&MS to discriminate between clinical and community samples was assessed using mean comparisons, receiver operating curves (ROC) and comparing proportions above the scales’ clinical thresholds. In the third stage correlations between the M&MS and Parent SDQ and SDQ self-report were explored to assess inter-rater reliability and construct validity. Lastly, the predictive validity of the *emotional difficulties* and *behavioural difficulties* scales was examined using clinical assessment.

## Results

### Internal reliability

Cronbach’s alpha for the two sub-scales in the clinical sample were good: emotional difficulties, α = .84, behavioural difficulties, α = .82. The reliabilities in the community sample were slightly lower: emotional difficulties, α = .77, behavioural difficulties, α = .77, which is similar to the internal reliabilities obtained in the community sample in the initial validation [9]. Comparatively, in the clinic sample, the internal reliabilities were slightly lower for both the self-report SDQ (emotional symptoms, α = .83, conduct problems, α = .75) and parent completed SDQ (emotional symptoms, α = .80, conduct problems, α = .76).

### Discriminating between clinic and community samples

As can be seen in Table 1 mean scores on both the scales were significantly higher in the clinic sample when compared to the community sample. For the emotional difficulties scale, on average there was a difference of more than 4-points on the scale (t (167.49) = -7.87, p < 0.001,) and for the behavioural difficulties scale an average difference of 2.7 points on the scale (t (166.95) = -7.58, p < 0.001,).

**Table 1:** Comparisons between the clinic and community samples for the emotional and behavioural difficulties scales

**Scale**	**Sample**	**Mean (SD)**	**Mean comparisons**	**Area under the curve (95% CI)**
*Emotional difficulties*	Clinic	8.65 (4.06)	t = -7.87***	.79 (.73-.86)
	Community	4.40 (3.14)	d = 1.17	
*Behavioural difficulties*	Clinic	5.13 (2.74)	t = -7.58***	.78 (.71-.84)
	Community	2.42 (2.05)	d = 1.12	

*Note.* ***p < .001.

To estimate the ability of the measure to discriminate between the community and clinical sample ROC analysis was conducted for both scales. ROC curves are based on statistical decision theory and demonstrate the ability of a test to discriminate between alternative states of health [18], in this case mental health. The main statistic, the area under the curve (AUC), represents the probability that the measure will discriminate a positive (clinical/high-risk) case from a negative (community/low-risk) case. The AUC statistic for the *emotional difficulties* scale was .79 (SE = .03) and for the *behavioural difficulties* scale was .78 (SE = .03).

In terms of participants having scores higher than the threshold score for problems, on the *emotional difficulties* scale 40% of the clinic sample scored above the threshold whereas 8.8% of the community sample scored above threshold (Odds Ratio [OR] = 6.92, 95% CI = 2.99-16.01). On the *behavioural difficulties* scale 41% of the clinical sample had above threshold scores as compared to 6.6% of the community sample (OR = 9.71, 95% CI = 3.84-24.54). Overall, 58% of the clinic sample and 12% of the community sample had an above threshold score in either scale which represents overall sensitivity of the measure to individuals with risk (OR = 10.14, 95% CI = 4.77-21.59).

### Correlations with parent SDQ and SDQ self-report

Table 2 presents the correlations between the emotional and behavioural scales of the M&MS, Parent SDQ and SDQ self-report. The correlations between the corresponding scales of the parent SDQ and the M&MS were both 0.30 and significant at p < 0.001.

**Table 2:** Correlations between M&MS, parent SDQ and SDQ self-report

**Variable**	**1.**	**2.**	**3.**	**4.**	**5.**
1. M&MS emotional difficulties	-				
2. M&MS behavioural difficulties	.12	-			
3. Parent SDQ emotional symptoms	.30**	.1	-		
4. Parent SDQ conduct problems	-.27*	.30**	.08	-	
5. SDQ self-report emotional symptoms	.85***	.11	.41**	-.17	-
6. SDQ self-report conduct problems	-.07	.56***	.01	.46**	-.18

*p < .05, **p < .01, ***p < .001. *Note.* Sample size for M&MS – Parent SDQ assessments N = 82-83, M&MS – SDQ-SR N = 52-53, Parent SDQ – SDQ-SR N = 48.

In terms of correlation with the self-report version of the SDQ, completed by only 11+ year old participants, the corresponding emotional scales correlated highly (r = .85), and the behaviour scales had moderately high correlations (r = .56). The non-corresponding scales had very low correlations (.11 and -.07), which are comparable to the correlations between non-corresponding scales of the M&MS (r = .12) and SDQ-self-report (r = -.18).

### Sensitivity to clinical assessment

A descriptive approach was used to explore the sensitivity of the *emotional difficulties* and *behavioural difficulties* scales in relation to clinical assessment as a way of illustrating the clinical utility and interpretability of the two sub-scales. Table 3 presents means and proportions above the sub-scale thresholds for each of the clinical assessment groupings (emotional, behavioural, emotional and behavioural, other), individuals assigned ICD Z-scores and individuals without diagnosis or presenting problems.

**Table 3:** Emotional difficulties and behavioural difficulties scales by clinical assessment

**Clinical assessment grouping (N)**	**Emotional difficulties scale**		**Behavioural difficulties scale**	
	**Mean (SD)**	**% Above threshold (N)**	**Mean (SD)**	**% Above threshold (N)**
Emotional (34)	10.76(4.13)	65 (22)	5.16 (2.48)	41 (14)
Behavioural (7)	4.67 (2.65)	0 (0)	4.57 (2.23)	29 (2)
Emotional/Behavioural(13)	8.08 (4.95)	31 (4)	7.00 (2.61)	77 (10)
Other (25)	7.76 (3.43)	32 (8)	4.61 (2.99)	32 (8)
Z-code (30)	8.09 (3.85)	37 (11)	4.77 (2.73)	43 (13)
No Diagnoses (7)	7.00 (2.61)	14 (1)	6.49 (2.20)	57 (4)

Individuals with emotional related problems had high scores on the *emotional difficulties scale* with more than 65% having scores above the clinical threshold. In terms of the *behavioural difficulties scale* there was a discrepancy between individuals assessed as having just behavioural symptoms and those with co-morbid emotional and behavioural symptoms in terms of their reporting of behavioural symptoms. A much smaller proportion of those assessed as having only behavioural problems scored above the threshold on the behavioural difficulties scale (29%) in comparison to those with comorbid emotional and behavioural problems (77%).

## Discussion

The M&MS measure is a recently developed measure that has been widely used as part of a national evaluation of school-based mental health support in England (40,000 plus young people [19]). Although Deighton et al. [9] established the measure’s properties in a community sample, additional analyses of the measure’s capacity to discriminate between high-risk and low-risk samples was a necessary step to justify its use as a self-report screening tool for mental health problems in community settings. This is especially relevant as the measure is currently being introduced as part of a UK national initiative to improve quality of psychological therapies and a programme promoting use of outcome monitoring in child and adolescent mental health services in the UK [20]. There is increasing emphasis on user perspective and patient-reported outcomes measures [21]. However, to date this has been more problematic for younger populations, with almost all data being provided by adult proxies on their behalf [22]. With further development, this measure has the potential to fill the gap for a free-to-use, brief, self-report measure that extends into this pre-adolescent age range in both community and clinic settings. The main benefit of examining the transferability of the measure from community to clinic settings is that it provides practitioners a simple self-report tool to help assess clinical need.

Analyses indicate that both the scales, *emotional difficulties* and *behavioural difficulties,* of the M&MS questionnaire sufficiently discriminate between a clinic (high-risk) and community (low-risk) sample. The amount of discrimination as represented by the AUC statistics (*emotional difficulties* = .79, *behavioural difficulties* = .77) are comparable to the AUC of the emotional symptoms scale (.75) and the conduct problems scale (.77) of the self-report SDQ [16]. In mental health in particular, very high scores for discrimination (e.g., >.8) are rare, partly because of the overlap in characteristics of community and clinical populations. Specifically, for mental health problems, being in a community sample does not indicate the absence of clinical problems and, correspondingly, a substantial proportion of young people who attend mental health services have no impairment or diagnosis [23]. Mean differences between the clinic and community sample were statistically significant with large effect sizes (d > 1.1) with individuals in the clinic sample being 4.5 times more likely to be above the threshold indicating problems on the *emotional difficulties* and 6 times more likely to be above the threshold indicating problems on the *behavioural difficulties* scale. Sensitivity of the individual scales of M&MS was 40-41%, The overall sensitivity of the scales’ thresholds was 58%, which is comparable to the 59% found for the SDQ [16]. This suggests that even though the M&MS is a brief measure with a general mental health focus it captures clinical need to a similar level as other brief measures such as the SDQ. In terms of the community sample 12% had scores above the threshold, which is lower than the 23% found by Goodman et al. [16] for the SDQ but is more in accordance with the 9-12% expected from a normal representative population in this age range [13,24].

The measure has good internal reliability as indicated by the Cronbach’s alphas of the two sub-scales (*emotional difficulties*, α = .84; *behavioural difficulties*, α = .82). The correlations between the corresponding scales of the M&MS and self-rated SDQ were high (*emotional difficulties* r = .85; *behavioural difficulties*, r = .56) and compared favourably to the correlations found in community samples (*emotional difficulties* r = .67; *behavioural difficulties* r = .7 [9]) which supports the measure’s construct validity in the clinic setting. Correlations between the corresponding scales of the self-reported measures and the parent SDQ were similar for M&MS with the parent SDQ (.3) and the self-report SDQ with the parent SDQ (.4). Overall the inter-rater correlations for the M&MS were in line with expected correlations between parent and child report which are generally significant but not high and were comparable to results from other measures (average r = .25) found in a meta-analysis [25].

Cross-referencing clinical assessment with the scale scores provides some evidence that both scales are responsive to clinical diagnoses as indicated by the mean scores and proportion above the clinical threshold in each diagnostic group. Given the numbers are small this could be a chance observation but the finding suggests that children with only a behavioural assessment might have more difficulties perceiving problems with their own behaviour. Alternatively the measure might not be effective at capturing self-reported difficulties in this particular sub-population. Additional research is required specifically exploring this discrepancy in self-reporting behavioural problems.

While clinical assessments provide early indication of the scales’ sensitivity to case type, the small numbers identified within each diagnostic category mean that formal statistical testing could not be carried out. This is something that could be explored in further studies when the measure is used more widely with clinic samples. Of particular interest is the consideration of an amendment to the clinical thresholds of the *emotional difficulties* scale. In the initial validation [9], thresholds were computed using an equi-percentile approach with the SDQ in the community sample. The results of this study indicate that a lower threshold might capture clinical levels of emotional problems better. This could be compounded by the use of thresholds developed on the computer-based survey, as children have been shown to report less problems on the paper based survey of the questionnaire [15], suggesting the need for different norms and thresholds for the paper survey. Future research should explore this possibility to ensure the measure has optimum screening capability.

## Conclusion

The primary aim of the current paper was to establish the credentials of M&MS as a screening tool for use in community settings. As such, results indicate that the measure discriminates sufficiently between clinic and community samples. However, the measure requires further research regarding responsiveness to change over time.

In conclusion, the findings of this study indicate that this measure sufficiently discriminates between high-risk (clinic) and low-risk (community) samples, has good internal reliability, compares favourably with existing self-report measures of mental health and has comparable levels of agreement between parent-report and self-report to other measures. Alongside existing validation of the M&MS [9,15], these findings justify the measures use as a self-report screening tool for mental health problems in community settings. Hence, the measure fills the gap for a free, brief self-report measure for under 11 year olds that can be used in community settings to identify children with mental health difficulties to facilitate them receiving the right support and intervention.

## Competing interests

The authors have no competing interests to declare with regards to this paper.

## Authors’ contributions

All authors contributed to the conception of this work, were involved in drafting and revising the content and approved it for publication.
